# Obsessive-compulsive symptoms relating to psychosocial functioning for people with schizophrenia, schizoaffective disorder, or bipolar disorder

**DOI:** 10.1017/neu.2024.42

**Published:** 2024-10-10

**Authors:** Nina Grootendorst-van Mil, Chin-Kuo Chang, David Chandran, Frederike Schirmbeck, Nico van Beveren, Hitesh Shetty, Robert Stewart, Deborah Ahn-Robbins, Lieuwe de Haan, Richard D Hayes

**Affiliations:** 1 Department of Psychiatry, Erasmus MC University Medical Center, Rotterdam, The Netherlands; 2 Antes Center for Mental Health Care, Rotterdam, The Netherlands; 3 Global Health Program, College of Public Health, National Taiwan University, Taipei City, Taiwan; 4 Institute of Epidemiology and Preventive Medicine, College of Public Health, National Taiwan University, Taipei City, Taiwan; 5 Institute of Psychiatry, Psychology and Neuroscience, King’s College London, London, UK; 6 South London and Maudsley NHS Foundation Trust, London, UK; 7 Department of Psychiatry, Amsterdam UMC Location AMC, Amsterdam, The Netherlands; 8 Arkin Institute for Mental Health, Amsterdam, The Netherlands

**Keywords:** schizophrenia, schizoaffective disorder, bipolar disorder, obsessive-compulsive disorder, psychosocial functioning

## Abstract

To assess the psychosocial functioning concerning obsessive-compulsive symptoms (OCS) and/or obsessive-compulsive disorder (OCD) comorbidity in people with schizophrenia, schizoaffective disorder, or bipolar disorder diagnosed in a large case register database in Southeast London. Data were retrieved from the South London and Maudsley NHS Foundation Trust Biomedical Research Centre (SLaM BRC) register using Clinical Record Interactive Search (CRIS) system, a platform allowing research on full but de-identified electronic health records for secondary and tertiary mental healthcare services. Information of schizophrenia, schizoaffective disorder, bipolar disorder diagnosis and OCS/OCD status was ascertained from structural or free-text fields through natural language processing (NLP) algorithms based on artificial intelligence techniques during the observation window of January 2007 to December 2016. Associations between comorbid OCS/OCD and recorded Health of the Nation Outcome Scales (HoNOS) for problems with activities of daily living (ADLs), living conditions, occupational and recreational activities, and relationships were estimated by logistic regression with socio-demographic confounders controlled. Of 15,412 subjects diagnosed with schizophrenia, schizoaffective disorder, or bipolar disorder, 2,358 (15.3%) experienced OCS without OCD, and 2,586 (16.8%) had OCD recorded. The presence of OCS/OCD was associated with more problems with relationships (adj.OR = 1.34, 95% CI: 1.25–1.44), ADLs (adj.OR = 1.31, 95%CI: 1.22–1.41), and living conditions (adj.OR = 1.31, 95% CI: 1.22–1.41). Sensitivity analysis revealed similar outcomes. Comorbid OCS/OCD was associated with poorer psychosocial functioning in people with schizophrenia, schizoaffective disorder, or bipolar disorder. This finding highlights the importance of identification and treatment of comorbid OCS among this vulnerable patient group.

## Significant Outcomes

- During eight-year observation, 15,412 people of schizophrenia, schizoaffective disorder, or bipolar disorder in Southeast London revealed a substantial portion of 32.1% (*n* = 4,944) having had obsessive-compulsive symptoms (OCS), including 2,586 subjects with a diagnosis of obsessive-compulsive disorder (OCD).
- After confounding control, the presence of either OCS or OCD was significantly associated with problems with relationships, activities of daily living (ADLs), living conditions, and problems of occupation and activities, compared to the group with neither OCS nor OCD.
- When dividing the OCS/OCD status into alternative categories, both OCS (but not OCD) and OCD groups were found more likely to have problems with relationships, ADLs, and living conditions, compared to the reference group after confounding adjustment.

## Limitations

- Classification of OCS and OCD status was based on unprompted documentation in health records in current analyses, not actively collected for research purposes for the practical difficulties faced by clinicians in judging obsession from delusional thought content.
- Health of the Nation Outcome Scales (HoNOS) was not available for all the subjects, for which we cannot rule out the possibility of selective missing.
- With a cross-sectional design, patients with OCS/OCD comorbidity might clinically experience fluctuations and temporary remission of OCS symptoms, without detailed temporality of the occurrence of OCS and functional problems detected for further analysis.

## Introduction

Schizophrenia, schizoaffective disorder, and bipolar disorder affect many aspects of an individual’s ability to function in daily life. However, there is a certain level of variety in symptomatic profile and severity within and between these disorders, resulting in substantial heterogeneity in psychosocial outcomes (Switaj *et al*., 2012). Identifying characteristics predicting psychosocial outcomes is critical to developing and evaluating interventions to reduce longer-term disadvantages. Over the years, obsessive-compulsive symptoms (OCS) have been described for schizophrenia in various forms as a part of symptoms occurring in people with schizophrenia (Zink, 2014).

A meta-analysis summarising 43 studies, including nearly 4,000 individuals with schizophrenia or schizoaffective disorder, estimated the prevalence of obsessive-compulsive disorder (OCD) to be around 13% (Swets *et al*., 2014). In bipolar disorder, the prevalence of comorbid OCD was also found to be substantial, ranging 13–23% (Cederlof *et al*., 2015). In the current study population, we obtained comparable prevalence using natural language processing (NLP) techniques to support automated, text-based ascertainment of recorded OCS and/or OCD in electronic clinical records from a large UK mental health case registry (Ahn-Robbins *et al*., 2022). In contrast, the lifetime prevalence of OCD is approximately 2% in the general population (Ruscio *et al*., 2010). OCS, defined as an obsession or compulsion, were observed in 30% of the people diagnosed with schizophrenia spectrum disorders (Swets *et al*., 2014).

The meta-analysis performed by Cunill and colleagues reported higher positive and negative symptom severity in patients with schizophrenia, if OCS were present (Cunill *et al*., 2009). However, other studies revealed inconsistent findings, where some did not find significant associations (Poyurovsky *et al*., 2008; Faragian *et al*., 2009; Nasrollahi *et al*., 2012; de Haan *et al*., 2013), and some others observed fewer positive or negative symptoms in people with schizophrenia and co-existing OCS/OCD, instead (Poyurovsky *et al*., 1999; Tibbo *et al*., 2000; de Haan *et al*., 2005). In most reported studies, people with schizophrenia and comorbid OCS show worse functioning than those without OCS (Fenton and McGlashan, 1986; Lysaker *et al*., 2004; Ongur and Goff, 2005; Guillem *et al*., 2009; Hwang *et al*., 2009; Schirmbeck *et al*., 2018). In bipolar disorder, the presence of comorbid OCD was associated with poorer functioning and poorer quality of life in physical, psychological, social, and environmental domains in a systematic review summarising the results of seven studies (Amerio *et al*., 2014). Previous research, however, has been limited by the use of widely differing diagnostic tools and symptom-rating instruments, potentially generating heterogeneous results or findings with restricted generalisability. More importantly, selective non-response, dropout, and missing outcomes are potential sources of biases or insufficient representation of groups at a higher risk (Hunter and Lysaker, 2015).

Manually conducted audits are limited by the number of cases which can be reasonably examined. Case registers have been enhanced through the digitalisation and mass archiving of health records, creating large and generalisable samples, as well as through advanced technique development in automated text-based searches of clinical records, providing greater breadth and depth of information (Lin *et al*., 2013; Shivade *et al*., 2014).

### Aims of the study

In the current study, we used NLP techniques to examine the hypothesis that existence of obsessive-compulsive symptoms and/or OCD is associated with worse functional status, as indicated by difficulties with relationships, activities of daily living (ADL), living conditions, and occupational/recreational activities in people with schizophrenia, schizoaffective disorder, or bipolar disorder.

## Methods

### Setting and study population

The study was conducted using clinical data from the electronic health records of people receiving secondary mental healthcare from the South London and Maudsley NHS Foundation Trust (SLaM). SLaM has a near-monopoly provision of mental health services to a defined geographic catchment of approximately 1.36 million residents of Lambeth, Southwark, Croydon, and Lewisham in south London, UK. SLaM service encompasses all aspects of secondary mental healthcare across all age groups, including inpatient, community, general hospital liaison, and forensic services, as well as tertiary national specialist services. Electronic clinical records have been comprehensively filed across all SLaM services since 2006. The Clinical Record Interactive Search (CRIS) system, supported by SLaM’s National Institute of Health Research (NIHR) Biomedical Research Centre (BRC), was developed in 2008 to enable researchers to search and retrieve de-identified data from the electronic clinical records efficiently for research purposes. The protocol for this case register has been described in detail elsewhere (Perera *et al*., 2016), with currently over 350,000 service users represented in CRIS. The authors assert that all procedures contributing to this work comply with the ethical standards of the relevant national and institutional committees on human experimentation and with the Helsinki Declaration of 1975, as revised in 2008. All procedures involving human subjects/patients were approved as an anonymised data resource for secondary analyses obtained from Oxfordshire Research Ethics Committee (reference number: 18/SC/0372). According to the regulations for anonymised data analysis, no informed consent is required for secondary data analyses with the choice of ‘opt out’ given to the members of CRIS.

Individuals who had received a diagnosis of schizophrenia (ICD-10 code: F20), bipolar disorder (F31), or schizoaffective disorder (F25) during the specific observation period (from 1 January 2007 to 31 December 2016, inclusive) and who had been assessed by clinicians using the Health of the Nations Outcome Scale (HoNOS) (Wing *et al*., 1998; Wing *et al*., 1999) at least once during this observation period were included in this dynamic cohort that also was described before by our study team (Ahn-Robbins *et al*., 2022). Both psychotic spectrum disorders (such as schizophrenia and schizoaffective disorder) and bipolar disorder, though clinically distinct, share overlapping neurobiological pathways that may predispose individuals to comorbid conditions such as OCS/OCD. Supported by recent research, this overlap reveals common genetic, cognitive, and functional impairments (Hamidian *et al*, 2020; Chen *et al*, 2023). By analysing these disorders together, we can conduct a comprehensive examination across a broad spectrum of severe mental health conditions, which enhances the generalisability of our findings and aids in identifying potential common therapeutic targets.

### Assessment of obsessive-compulsive symptoms

NLP algorithms allow the automated extraction and coding of information from unstructured clinical text records (free-text fields, including recorded patient reviews and clinical correspondence), which would not otherwise be available in structural fields. These algorithms have been widely developed and applied in CRIS to enhance the depth of data mining for analyses at scale (Perera *et al*., 2016). We developed NLP algorithms specifically to extract documentation of obsessive-compulsive symptoms (OCS) or OCD from free-text fields in clinical assessments and correspondences in CRIS, using General Architecture for Text Engineering (GATE) software (Cunningham *et al*., 2013). Full details concerning the development of this NLP application and the criteria for identifying OCS/OCD were described elsewhere by Chandran et al. (Chandran *et al*., 2019). In brief, coding rules to determine the presence of OCS, including OCD, in clinical assessments and correspondences were based on the Yale-Brown Obsessive Compulsive Scale (Y-BOCS) as a guideline for development (Goodman *et al*., 1989). The OCS algorithm was able to identify OCS, including OCD, with a precision (i.e., positive predictive value) of 0.64 and a recall (i.e., sensitivity) of 0.76, and instances of a recorded clinical diagnosis of OCD were identified with a precision/recall of 1.00/0.86. In addition, the application was supplemented with output from a pre-existing NLP algorithm ascertaining texts associated with diagnosis statements, performed at the precision/recall of 0.98/0.88 for OCD (ICD10 code F42) (Perera *et al*., 2016). Data from the algorithms of OCS, psychiatric diagnosis, and structured fields in CRIS were combined to produce the outcome measure of OCS, including OCD.

### Assessment of functional status

Functional status was measured with the Health of the Nation Outcome Scale (HoNOS), which is a clinical outcome measure in routine use across mental health services in the UK (Wing *et al*., 1998). If multiple HoNOS assessments were available, the assessment closest to the first schizophrenia, schizoaffective disorder, or bipolar disorder diagnosis was chosen. Clinicians rate patients in 12 domains on a 5-point severity scale ranging from ‘no problem’ to ‘very severe problem’. A lower score indicates fewer problems or better psychosocial functioning. In the current study, the primary outcomes of interest were the HoNOS items which address function across four domains: i) Item 9 assesses problems with social relationships, including active or passive withdrawal from social relationships, and/or non-supportive, destructive or self-damaging relationships; ii) Item 10 assesses limitations in ADLs, including problems with basic activities of self-care (e.g., eating, washing, dressing, and toilet), as well as more complex skills, like budgeting, shopping, and use of transport; iii) Item 11 assesses sub-optimal living conditions, in particular, whether basic necessities (heat, light, and hygiene) are met; iv) Item 12 assesses problems with occupational and recreational activities, including whether there is help to cope with disabilities, and opportunities for maintaining or improving occupational and recreational skills and activities. The five response options of all HoNOS items were dichotomised by grouping 0 (no problem) and 1–4 (minor problem to significant problem). The validity and feasibility of applying the HoNOS in a variety of patient groups have been assessed in a number of previous studies (Orrell *et al*., 1999; Pirkis *et al*., 2005; Hunter *et al*., 2009).

### Socio-demographic and clinical characteristics

Study subjects were grouped into diagnostic categories based on the mental disorder diagnosis received before or during the observation period. Information on diagnosis came from both the NLP algorithm, as previously described, and from recorded structured diagnostic fields, which apply ICD-10 codes. Where study subjects had received more than one diagnosis of interest during the observation period, the diagnosis was hierarchically assigned as schizophrenia, schizoaffective disorder, and then bipolar disorder.

Other variables included secondary comorbid diagnoses of alcohol use disorder (F11), opioid use disorder (F10), and major depressive disorder (F33) that were diagnosed before or during the observation period. These comorbidities were also extracted from structured fields and free-text fields using the psychiatric diagnosis application of GATE described above.

Data on age, sex, ethnicity, marital status, socio-economic status in small residing regions, and medication use were defined from routinely completed fields. Age was calculated at the date on which the individual received their first schizophrenia, schizoaffective disorder, or bipolar disorder diagnosis during the observation period. Ethnicity was classified into the following groups: ‘White British’, ‘Other white’, ’South Asian’, ‘East Asian’, ‘Caribbean’, ‘Other black’, and ‘Others, mixed, and unknown’ according to categories defined by the UK Office for National Statistics. Marital status was taken from the last available entry and categorised as being in a relationship (including ‘married’, ‘civil partnership’, and ‘cohabiting’) and no relationship (including ‘divorced’, ‘civil partnership dissolved’, ’separated’, ’single’, ‘widowed’, and ‘unknown’).

Socio-economic status was measured using the Index of Multiple Deprivation score, which measures relative levels of deprivation in small-area neighbourhoods in the UK, given in percentiles (Department for Communities and Local Government, UK, 2019). The Index of Multiple Deprivation comprises seven deprivation domains taken from the 2011 UK census: ‘income’, ‘employment’, ‘health and disability’, ‘education skills and training’, ‘barriers to housing and services’, ‘crime’, and ‘living environment’. The address in England, recorded closest in time to the first schizophrenia, schizoaffective disorder, or bipolar disorder diagnosis received during the observation period, was used to obtain deprivation scores, with a separate category for homelessness. Information on physical illness or disability problems scores was also obtained from the closest HoNOS measurement (Wing *et al*., 1998).

### Statistical analysis

Descriptive statistics for covariates are presented as means ± SD for continuous variables and frequencies with percentages for categorical variables. We chose a hierarchical approach to reduce the risk of type I error and performed an initial overall analysis to examine the association between either OCS or OCD (i.e., the broadest definition) versus neither with psychosocial functioning measured with problem-indicative scores on the four HoNOS items as outcomes by logistic regression models (first level). If any association between OCS/OCD comorbidity with psychosocial functioning in this overall analysis was observed, associations between the alternative groups of OCS (but no OCD) and just OCD (i.e., the narrowest definition) on functional problems, with rest of people as the reference group in analyses (second level) were tested. Potential socio-demographic and clinical confounders were subsequently included in the analyses as covariates. The socio-demographic and clinical variables adjusted for in our logistic regression models were selected based on previous literature, including their well-documented influence on psychosocial outcomes (Ruscio *et al*., 2010; Fontenelle *et al.*, 2006), and/or the likelihood that they may be associated with the presence of OCD/OCS and have an impact on psychosocial outcomes. These included age, sex, ethnicity, socio-economic status, psychiatric comorbidities of depression and anxiety, and medication use. This approach improved the likelihood that the observed associations could be robust and valid.

The decision to combine individuals with diagnoses of schizophrenia, schizoaffective disorder, and bipolar disorder (i.e., SMI diagnoses) into one analysis was informed be their overlapping psychopathological features, suggesting a comparable impact of OCS/OCD on psychosocial functioning. However, we acknowledge the possibility that there may be differences across diagnostic groups with regard to the impact of OCS on the outcomes investigated. We performed a test for interaction to determine if there was evidence of effect modification by SMI diagnosis with a plan to stratify by SMI diagnosis if this test indicated there was evidence of effect modification.

In addition, we tested an alternative cut-off for dichotomising the HoNOS response options (0-1: no problem or minor problem, compared to 2–4: significant problem) as sensitivity analysis.

## Results

**Figure 1.**
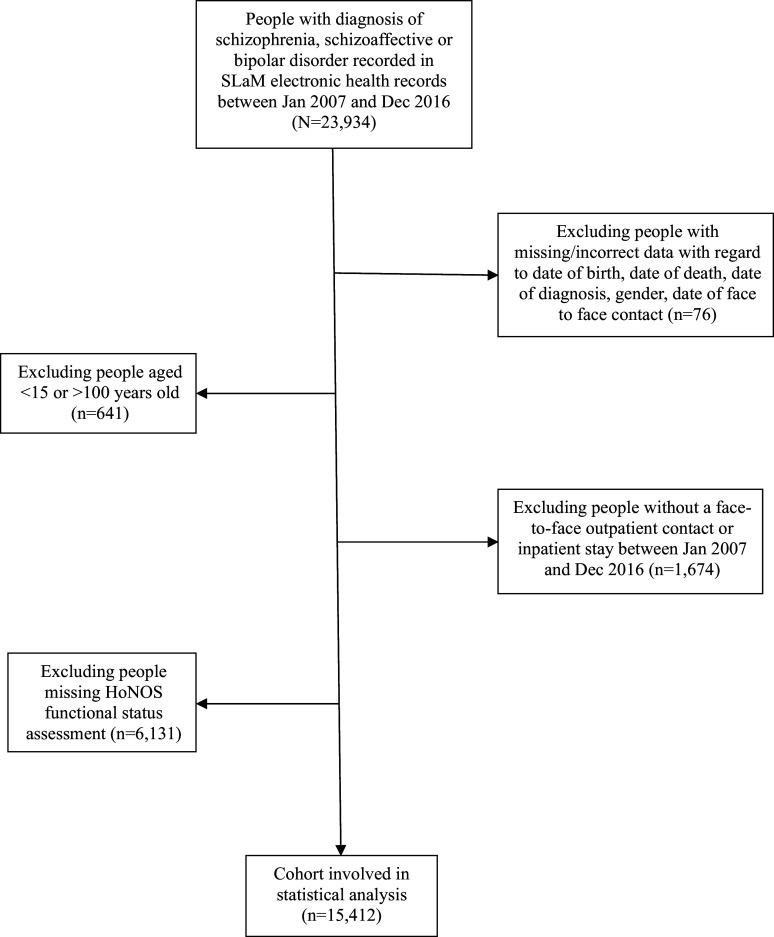
Flow chart of the people with a diagnosis of schizophrenia, schizoaffective, or bipolar disorder in South London and maudsley NHS foundation trust from 2007 to 2016 for the use of electronic health records in data mining.

Of the 23,934 individuals with a diagnosis of schizophrenia, schizoaffective disorder, or bipolar disorder during the eight-year observation window, 15,412 (64.4%) members with information on functional status outcomes were included in the analysed sample (Figure 1). Socio-demographic and clinical characteristics are presented by OCS/OCD status in Table 1. Of the study population, 4,944 (32.1%) individuals were recorded as having OCS (including *n* = 2,586 with a clinical diagnosis of OCD).

**Table 1.** Characteristics of the four comparison groups of OCS and/or OCD status among patients with schizophrenia, schizoaffective disorder, or bipolar disorder (*N* = 15,412)

Variables	OCS/OCD status Mean ± SD / Number (% in column)			
	Neither (*n* = 10,468)	Either OCS or OCD (*n* = 4,944)	OCS but no OCD (*n* = 2,358)	OCD (*n* = 2,586)
Age (years old)	42.38 ± 15.24	3,8.86 ± 13.57	41.82 ± 14.42	36.17 ± 12.80
15–29	2,474 (23.6%)	1,529 (30.9%)	557 (23.6%)	972 (37.6%)
30–39	2,654 (25.4%)	1,212 (24.5%)	526 (22.3%)	686 (26.5%)
40–49	2,487 (23.8%)	1,196 (24.2%)	656 (27.8%)	540 (20.9%)
50 and over	2,853 (27.3%)	1,007 (20.4%)	619 (26.3%)	388 (15.0%)
Gender				
Female	4,913 (46.9%)	2,257 (45.7%)	1,091 (46.3%)	1,166 (45.1%)
Male	5,555 (53.1%)	1,347 (54.3%)	1,267 (53.7%)	1,420 (54.9%)
Ethnicity				
White	4,991 (47.7%)	2,444 (49.45%)	1,063 (46.4%)	1,381 (53.4%)
Asian	660 (6.0%)	2,86 (5.8%)	122 (5.2%)	164 (6.3%)
Black	3594 (34.3%)	1,737 (35.1%)	954 (40.5%)	783 (30.3%)
Others/mixed/unknown	1,223 (11.7%)	447 (9.0%)	189 (8.0%)	258 (10.0%)
Married or cohabiting				
No	8,844 (84.5%)	4,441 (89.8%)	2,144 (90.9%)	2,297 (88.8%)
Yes	1,624 (15.5%)	503 (10.2%)	214 (9.1%)	289 (11.2%)
Multiple deprivation score in the area of residence				
1^st^ tertile	3,319 (31.7%)	1,579 (31.9%)	684 (29.0%)	895 (34.6%)
2^nd^ tertile	3,408 (32.6%)	1,423 (30.8%)	740 (31.4%)	783 (30.3%)
3^rd^ tertile	3,295 (31.5%)	1,636 (33.1%)	830 (35.2%)	806 (31.2%)
Homeless/address missed	446 (4.3%)	206 (4.2%)	104 (4.4%)	102 (3.9%)
Psychiatric diagnosis				
Schizophrenia	6,547 (62.5%)	3,182 (64.4%)	1,566 (66.4%)	1,616 (62.5%)
Schizoaffective disorder	683 (6.5%)	364 (7.4%)	186 (7.9%)	178 (6.9%)
Bipolar disorder	3,238 (30.9%)	1,398 (28.3%)	606 (25.7%)	792 (30.6%)
Comorbid alcohol use disorder				
No	9,917 (94.7%)	4,559 (92.2%)	2,181 (92.5%)	2,378 (92.0%)
Yes	551 (5.3%)	385 (7.8%)	177 (7.5%)	208 (8.4%)
Comorbid opioid use disorder				
No	10,299 (98.4%)	4,817 (97.4%)	2,301 (97.6%)	2,516 (97.3%)
Yes	169 (1.61%)	127 (2.6%)	57 (2.4%)	70 (2.7%)
Number of physical illness or disability problems				
0	6,611 (63.2%)	3,119 (63.1%)	1,394 (59.3%)	1,725 (66.8%)
1	1,756 (16.8%)	859 (17.4%)	437 (18.6%)	422 (16.3%)
2	1,230 (11.8%)	616 (12.5%)	335 (14.3%)	281 (10.9%)
3	655 (6.3%)	281 (5.7%)	152 (6.5%)	129 (5.0%)
4 or more	206 (2.0%)	56 (1.1%)	31 (1.3%)	25 (1.0%)

Logistic regression analyses showed that, before and after confounding control, the presence of either OCS or OCD was associated with problems with relationships, ADLs, living conditions, and problems of occupation and activities, compared to the group with neither OCS nor OCD (Table 2). When we divided the exposure of interest into the alternative exposure categories, both OCS (but not OCD) and OCD were found more likely to have problems with relationships, ADLs, and living conditions, compared to the reference group, before and after confounding adjustment. Although similar results were obtained, overall effect estimates seemed slightly larger for OCS than OCD. Associations between OCS (but not OCD) and OCD with problems of occupation and activities lost statistical significance after adjustment for potential confounders. Similar results were observed if the alternative cut-off points of the HoNOS scores were used (details not shown). The test for interaction indicated no evidence of effect modification by SMI diagnosis. Consequently, we present the results with the SMI diagnoses combined.

**Table 2.** Associations between comorbid OCS/OCD status and outcome measures of functioning in people with schizophrenia, schizoaffective disorder, or bipolar disorder served by SLaM services in univariable and multivariable logistic regression models (*N* = 15,412)

OCS/OCD status	Psychosocial Functioning Status							
	Problems with relationships		Problems with activity of daily living		Problems with living conditions		Problems with occupation and recreational activities	
	Crude OR (95% CI)	Adjusted OR (95% CI)^a^	Crude OR (95% CI)	Adjusted OR (95% CI)^a^	Crude OR (95% CI)	Adjusted OR (95% CI)^a^	Crude OR (95% CI)	Adjusted OR (95% CI)^a^
The rest of subjects	Reference	Reference	Reference	Reference	Reference	Reference	Reference	Reference
Either OCS or OCD	1.40 (1.31–1.51)	1.34 (1.25–1.44)	1.34 (1.25–1.44)	1.31 (1.22–1.41)	1.34 (1.25–1.44)	1.31 (1.22–1.41)	1.14 (1.07–1.22)	1.08 (1.01–1.16)
OCS but no OCD	1.50 (1.36–1.65)	1.45 (1.32–1.60)	1.53 (1.40–1.68)	1.43 (1.30–1.57)	1.53 (1.40–1.68)	1.43 (1.30–1.57)	1.16 (1.06–1.27)	1.08 (0.98–1.18)
OCD	1.32 (1.21–1.45)	1.24 (1.13–1.37)	1.19 (1.09–1.30)	1.21 (1.11–1.33)	1.19 (1.09–1.30)	1.21 (1.11–1.33)	1.12 (1.03–1.22)	1.09 (1.00–1.19)

a. Adjustment on age groups, sex, ethnicity, relationship, deprivation score, diagnosis of schizophrenia, schizoaffective disorder, or bipolar disorder, comorbidity of alcohol use disorder, comorbidity of heroin use disorder, use of antipsychotics or antidepressants, and physical illness or disability problems score in HoNOS.

## Discussion

### Summary of main findings

In a large study sample of people with schizophrenia, schizoaffective disorder, or bipolar disorder, we found that individuals with recorded OCS/OCD were more likely to have recorded difficulties with relationships, ADLs, and living conditions, compared to rest of the study sample. In the sample, the prevalence of either OCS or OCD (32.1%) was slightly higher than what was previously reported (13–23% in bipolar disorder and 30% in schizophrenia) (Swets *et al*., 2014; Cederlof *et al*., 2015). OCD itself represents a clinically heterogeneous disorder with a wide range of symptomatic variation and impairment. Our findings were in line with results from previous studies reporting poorer psychosocial functioning in schizophrenia, schizoaffective disorder, or bipolar disorder among individuals with comorbid OCS/OCD (de Haan *et al*., 2005; de Haan *et al*., 2013; Amerio *et al*., 2014). In the current study, associations between problems in psychosocial functioning and OCS (but no OCD) were slightly stronger than associations with OCD, differing from the hypothesis that an improving effect on function might be due to mild OCS (de Haan *et al*., 2013; Tonna *et al*., 2016). One possibility is that people recorded with OCD have received sufficient attention for the diagnosis to be considered and applied, therefore receiving treatments for their symptoms, while those with OCS may not be recognised and treated. Another possibility is that the presence of OCS in psychosis is associated with a syndrome of worse underlying severity, although this would require further empirical research.

### Relevance to existing literature

Previous findings indicated that half of the clozapine-treated patients with schizophrenia or schizoaffective disorder had been unrecognised for their comorbid OCD during routine psychiatric treatment (Mukhopadhaya *et al*., 2009). In terms of psychopathology, OCS comorbidity may have different clinical meanings in schizophrenia and bipolar disorder. OCS-related impairment in social functioning has been previously reported independent of negative and positive symptoms in people with schizophrenia (Hwang *et al*., 2009; Tonna *et al*., 2015). In a meta-analysis by Cunill and colleagues (Cunill *et al*., 2009), in patients with schizophrenia, no association between psychotic symptoms and OCS (using a categorical distinction between OCD vs. non-OCD) was found. As these authors concluded, a dimensional conceptualisation of the OC-schizophrenia spectrum might be preferable to the categorical approach. In bipolar disorder, OCSs are likely to follow the course of the disorder, typically with worsening during depression (78%) and improvement during (hypo)mania (64%). Compared to subjects without OCS/OCD, comorbid OCS/OCD status shows a more episodic course of OCS (up to 75% vs. 3%), as well as a doubled incidence of depressive episodes (Amerio *et al*., 2014; Ahn-Robbins *et al*., 2022). Previously, investigators disclosed mixed findings on the association between co-occurring OCS and impairment in social functioning. Lysaker et al. identified different subgroups of people with schizophrenia with comorbid OCS, reporting that lower levels of negative symptoms were associated with better social function in individuals with OCS comorbidity, whereas more cognitive impairment was associated with poorer social function (Lysaker *et al*., 2004). However, a more recent study could not replicate these findings (Swets *et al*., 2019). Other researchers proposed that a phase-dependent effect of OCS/OCD on the functional level, with a protective effect in the early stages of schizophrenia and greater impairment in chronic schizophrenia, may partially explain conflicting results on psychosocial functioning (Bottas *et al*., 2005; Tonna *et al*., 2015).

### Strengths and limitations

One of the strengths of the current study is the inclusion of a large sample of people with schizophrenia, schizoaffective disorder, or bipolar disorder and the ascertainment of recorded OCS/OCD using an innovative NLP algorithm applied to large volumes of clinical text. This approach enhances the representation of the clinical population of people suffering from schizophrenia, schizoaffective disorder, or bipolar disorder in a well-defined geographical catchment area, which increases the robustness and generalisability of our results. Text mining procedures in large databases representing large catchment areas may add valuable findings for translational research and help address earlier inconsistencies in the literature. Our research has limitations that should be borne in mind while interpreting our findings. First, the classification of OCS and OCD in our study was based on unprompted documentation in health records by healthcare professionals, which was not prepared for the purpose of research. Although the algorithms used to extract such statements achieved good precision and recall (Chandran *et al*., 2019), it is possible that we may have missed clinically relevant but unrecorded OCS/OCD. Moreover, the difficulty clinicians face in disentangling obsessive from delusional thought content could represent another reason for the potential misclassification of OCS/OCD (Berman *et al*., 1998). However, inaccuracies in the measurement of co-occurring OCS/OCD are most likely to have led to an underestimation of the true association with functional problems. Second, HoNOS was not available for all subjects, we cannot rule out this may have influenced our findings. Third, it has been noticed clinically that most patients with OCS/OCD comorbidity experience fluctuations and temporary remission of OCS symptoms (Schirmbeck *et al*., 2018). Consequently, in this study, it was challenging to delineate the temporality between the occurrence of OCS and functional problems, and the design and data availability restricted our ability to directly compare disease courses across different disorders. The dataset used primarily provided point-in-time assessments (in part due to limitations in the NLP application), which limits our ability to trace the development of OCS/OCD relative to the onset of psychotic symptoms. Additionally, we recognise the potential benefits of investigating the impact of bipolar mood states - including hypomania, euthymia, and hyperthymia - on primary outcomes in future research. Fourth, the high ethnic and social diversity within the SLaM catchment area suggests caution when generalising our findings to other urban and rural settings, both within the UK and internationally. The participants were sourced from secondary and tertiary mental healthcare services, which typically manage more severe or complex cases than those encountered in primary care or the broader community. While the study’s large sample size and use of NLP enhance its representativeness within London’s specific geographic area, extrapolating these results to broader populations should be done carefully. Finally, we report an observed association between relatively contemporaneous constructs without assuming causal relationships underlying these observations.

### Implications for future research and clinical practice

Comorbid OCS/OCD in schizophrenia, schizoaffective disorder, or bipolar disorder is common and associated with impairment in a number of domains of psychosocial functioning. Regardless of causal relationships, this suggests a vulnerable group for adverse outcomes that might benefit from focused intervention. Further research is required to establish whether interventions to improve OCS/OCD might contribute to achieving more positive psychosocial outcomes. Owing to the high prevalence of co-occurring OCS/OCD in schizophrenia, schizoaffective disorder, or bipolar disorder, the observed association with increased risks of functional problems is clinically meaningful. Current study demonstrates the feasibility and potential clinical and service applicability of automatically extracting psychiatric symptoms from text fields in electronic clinical records. Methodological development in characterising symptomatic phenotypes across large samples from data sources of electronic health records has potentially significant contributions to enhancing psychiatric research.
